# Ox-LDL Induces Dysfunction of Endothelial Progenitor Cells via Activation of NF-**κ**B

**DOI:** 10.1155/2015/175291

**Published:** 2015-03-04

**Authors:** Kang-ting Ji, Lu Qian, Jin-liang Nan, Yang-jing Xue, Su-qin Zhang, Guo-qiang Wang, Ri-peng Yin, Yong-jin Zhu, Lu-ping Wang, Jun Ma, Lian-ming Liao, Ji-fei Tang

**Affiliations:** ^1^Department of Cardiology, the Second Affiliated Hospital, Wenzhou Medical University, Wenzhou 325000, China; ^2^Academy of Integrative Medicine, Fujian University of Traditional Chinese Medicine, Fuzhou 350112, China

## Abstract

Dyslipidemia increases the risks for atherosclerosis in part by impairing endothelial integrity. Endothelial progenitor cells (EPCs) are thought to contribute to endothelial recovery after arterial injury. Oxidized low-density lipoprotein (ox-LDL) can induce EPC dysfunction, but the underlying mechanism is not well understood. Human EPCs were cultured in endothelial growth medium supplemented with VEGF (10 ng/mL) and bFGF (10 ng/mL). The cells were treated with ox-LDL (50 *µ*g/mL). EPC proliferation was assayed by using CCK8 kits. Expression and translocation of nuclear factor-kabba B (NF-*κ*B) were evaluated. The level of reactive oxygen species (ROS) in cells was measured using H2DCF-DA as a fluorescence probe. The activity of NADPH oxidase activity was determined by colorimetric assay. Ox-LDL significantly decreased the proliferation, migration, and adhesion capacity of EPCs, while significantly increased ROS production and NADPH oxidase expression. Ox-LDL induced NF-*κ*B P65 mRNA expression and translocation in EPCs. Thus ox-LDL can induce EPC dysfunction at least by increasing expression and translocation of NF-*κ*B P65 and NADPH oxidase activity, which represents a new mechanism of lipidemia-induced vascular injury.

## 1. Introduction

The activation of inflammatory signaling has been suggested to contribute to the development of atherosclerosis [1]. The transcriptional regulator nuclear factor-kabba B (NF-*κ*B) plays a central role in the inflammatory response [2, 3]. NF-*κ*B induces the expression of many inflammatory genes that encode mediators of atherogenesis, including inflammatory chemokines and adhesion molecules. Activation of NF-*κ*B has been detected in vascular smooth muscle cells (VSMCs) and macrophages and endothelial cells (ECs), which are involved in human atherosclerotic lesions [4–8]. Moreover, NF-*κ*B is involved in adherence of immune cells such as monocytes or lymphocytes to the vessel wall during atherosclerotic plaque formation [1]. Hence, NF-*κ*B pathway is strongly involved in the pathogenesis of cardiovascular diseases (CVDs).

A huge amount of evidence suggests that oxidized low-density lipoprotein (ox-LDL) contributes to atherogenesis [9, 10]. Endothelial cells exposed to ox-LDL secrete adhesion molecules, chemoattractant proteins, and colony-stimulating factors that enhance the infiltration, proliferation, and accumulation of monocytes/macrophages in the arterial wall [11–13]. When circulating ox-LDL is elevated, it represents an independent risk factor for acute cardiac events [14].

Endothelial regeneration plays an important role in restoring vascular tone in response to vascular injury. Mature endothelial cells have a very low regenerative capacity compared with circulating endothelial progenitor cells (EPCs), which can proliferate, migrate, and differentiate into mature EPs. EPCs have been shown to enhance the formation of new endothelium in animal models, in which vessel injury occurred after balloon injury, myocardial infarction, or heart transplantation [15, 16]. Thus, the presence of healthy EPCs is crucial to postevent vascular reconstruction [17, 18]. Not surprising, several groups have shown that ox-LDL is also detrimental to the growth and bioactivity of EPCs [19–23]. However, the underlying mechanism is still not well understood. In the present study, we investigated whether ox-LDL can cause activation of NF-*κ*B and the relationship between NF-*κ*B activation and oxidative stress in EPCs.

## 2. Materials and Methods

### 2.1. Cell Culture

Informed consent was obtained from all participating mothers. The study was approved by the Institutional Review Board of the Second Affiliated Hospital of Wenzhou Medical University. Umbilical cord blood (50 mL) from a normal delivery was collected by needle/syringe from the placental side of the umbilical vein after the newborn was delivered but prior to placental delivery. Cord blood mononuclear cells were isolated from umbilical cord blood by Ficoll gradient centrifugation. Then cells were seeded at 5 × 10^6^/cm^2^ into 6-well plates precoated with fibronectin (Roche Applied Science, Indianapolis, IN, USA). The culture medium was endothelial growth medium-2 (EGM-2; Lonza, Basel, Switzerland) supplemented with fetal calf serum (FCS, 10%, w/v) and antibiotics. Cells were cultured in a humidified incubator with 5% CO_2_ and initially allowed to adhere for 24 h, followed by medium change every 3 days. When cultures reached over 90% confluence, adherent cells were detached with 0.05% trypsin-EDTA (Gibco, Carlsbad, CA, USA) and replated.

### 2.2. Fluorescent Staining

After being cultured for 6 days, adherent EPCs were incubated with either 1,1′-dioctadecyl-1-3,3,3′,3′-tetramethyl-indo-carbocyanine perchlorate-acetylated-LDL (DiI-ac-LDL; 2.4 *μ*g/mL, Molecular Probe, USA) or rhodamine conjugated lectin (Lectin Kit, Sigma, USA) at 37°C for 4 hours and observed with an inverted fluorescent microscope (Leica, Wetzlar, Germany).

### 2.3. VIII-Related Antigen Immunohistochemistry

After cultured cells were fixed with 95% (v/v) alcohol for 30 min, 0.5% H_2_O_2_ in methanol (v/v) was added to inactivate endogenous peroxidase. Nonspecific background staining was blocked with goat serum for 20 minutes. The primary antibodies were applied in 1% bovine serum albumin (BSA) and the cells were then incubated for 60 minutes in a moist chamber at 25°C. After three washes in PBS, the slides were treated with secondary biotinylated antibody for 30 minutes. After three washes in PBS, strept actividin-biotin complex (SABC) liquid was applied for 20 minutes at 37°C according to standard protocol. Positive cells were stained brown. Gastric cancer cells (GSC7901) served as negative control.

### 2.4. Cell Proliferation Assay

Cell proliferation was quantified with CCK-8 method. Briefly, cells were plated in flat-bottomed 96-well microplates at 1 × 10^4^ cells/well and incubated in EGM-2 medium containing 2% CFS for 24 hours (6 wells per group). Then the culture medium was changed to EGM-2 medium containing 10% CFS and cultured for an additional 6 hours. Cells were finally exposed to ox-LDL for 18 hours. Control cells were without any treatment. CCK-8 (10 *μ*L/well) was added to the wells at the end of the experiment. After incubation at 37°C for 2 hours, the absorbance of each well was determined using a microplate reader at 450 nm. The degree of cell proliferation was determined as the percentage of absorbance of treated cells to that of control cells.

### 2.5. EPC Adhesion

EPCs (5 × 10^4^ cells/mL) were cultured in 96-well plates for 0.5 hour at 37°C in the presence or absence of ox-LDL. Nonadherent cells were removed by a thorough washing with PBS, and adherent cells were counted with an inverted microscope in 10 randomly selected vision fields (×400).

### 2.6. EPC Migration

EPCs were cultured for 24 hours at 37°C in the presence or absence of ox-LDL. Cells were harvested and adjusted to 5 × 10^4^ cells/mL. Cell migration was quantified by a transwell chemotaxis assay using a Boyden chamber [17]. Briefly, 2 × 10^4^ cells (150 *μ*L) were plated in the upper chamber. EBM-2 medium containing 10% FBS (100 *μ*L) was added to the lower chamber. The two chambers were separated by a membrane with 8 *μ*m pores (Corning Transwell). After 24 hours, the membranes were washed twice in D-Hank's (Gibco, USA) and were fixed in 4% formaldehyde. After wiping cells off the upper side of the membrane with a cotton swab (Q-tip), the membranes were detached and mounted on glass slides with 0.25% crystal violet. Migrated cells were counted with a microscopy. Each experiment was performed in triplicate, and the number of migrated cells was determined from 4 random 200x fields per membrane.

### 2.7. Real-Time RT-PCR Analysis

Total RNA was isolated and transcribed to cDNA using a RT-PCR kit (MBI Fermentas, Canada). RNA (1 *μ*g) was reverse transcribed in a total volume of 20 *μ*L. An aliquot of 1 *μ*L of the reverse transcription reaction was used in the real-time PCR reactions (20 *μ*L final volume) and performed in a Lightcycler 480 thermocycler (Roche). Fold inductions were calculated using the cycle threshold ΔΔCt method as previously described. PCR was performed at 95°C (30 s) followed by 40 cycles at 95°C (5 s)/60°C (20 s). SYBR green intercalating dye was used for signal detection. For each sample, the number of cycles required to generate a given threshold signal (Ct) was recorded. With a standard curve generated from serial dilutions of sample cDNA, the ratio of NF-*κ*B P65 expression relative to GAPDH expression was calculated for each experimental group and normalized to the control group. Sequences of the primers used in this study were as follows: P65 forward primer 5′-CAC CGG ATT GAG GAG AAA CGT-3′, reverse primer 5′-ATC TGC CCA GAA GGA AAC ACC-3′, GAPDH forward primer 5′-ATG GGG AAG GTG AAG GTC G-3′, reverse primer 5′-CTG GAA GAT GGT GAT GGG ATT-3′.

### 2.8. NF-*κ*B Translocation Assay

NF-*κ*B translocation in the cells was examined using a NF-*κ*B activation detection kit (Beyotime Institute of Biotechnology, Shanghai, China) which contains DAPI, anti-NF-*κ*B P65 mAb, and secondary Cy3-conjugated mAb dyes. EPCs were cultured for 24 hours at 37°C in the presence or absence of ox-LDL. Fixation, permeabilization, and immunofluorescence staining of the cells were performed according to the manufacturer's instructions. The difference between the intensity of nuclear and cytoplasmic NF-*κ*B-associated fluorescence was reported as translocation parameter.

### 2.9. Measurement of Intracellular ROS

The oxidation of 2′,7′-dichlorofluorescein diacetate (DCFH-DA, Sigma-Aldrich) to 2′,7′-dichlorofluorescein (DCF) was used to estimate the content of ROS [18]. EPCs in 24-well plates were grown in the presence or absence of ox-LDL for 24 hours. Thereafter, the cells were incubated with 5 *μ*mol/L CM-H2DCF-DA for 3 hours at 37°C. After removal of the medium and washing of the cells, the fluorescence intensity (relative fluorescence units) was measured at an excitation and emission wavelength of 488 nm and 522 nm, respectively, using a spectrofluorometer (Hitachi, Japan).

### 2.10. Quantitative Determination of NADPH by Colorimetric Analysis

NADPH oxidase activity was measured with a GENMED kit (Genmed Scientifics Inc., Shanghai, China) by colorimetric method. EPCs were plated (5 × 10^6^/mL) in six-well plates with EGM-2 medium containing 2% FBS. After 24 hours ox-LDL was added and cells were cultured for additional 24 h. Thereafter, the protein was extracted from cells and NADPH oxidase activity was determined according to the manufacturer's instruction.

### 2.11. Statistics

All data are presented as mean ± SEM. Comparisons between groups were made using one-way ANOVA. *P* < 0.05 was considered statistically significant.

## 3. Results

### 3.1. Isolation and Characterization of EPCs

**Figure 1:**
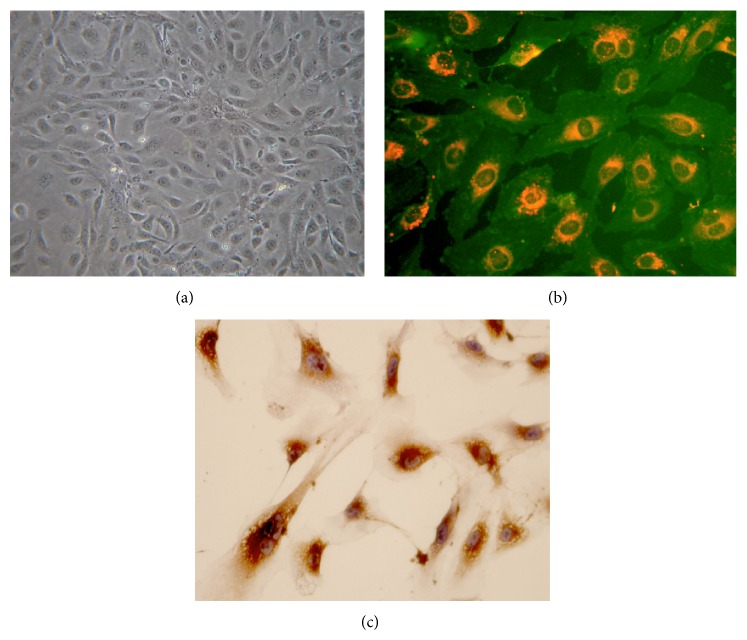
Identification of EPCs culture of MNCs for 7 days. (a) Cells had a typical cobblestone shape as shown by a phase-contrast inverted microscope. Images are visualized at ×200 magnification. (b) DiI-acetylated LDL uptake and fluorescein isothiocyanate (FITC)-UEA-1 binding and the merged image. Images are shown at ×400 magnification. Double-positive cells are yellow. (c) VIII-related antigen immunohistochemistry. Positive cells were stained brown.

Adherent cell clusters appeared after 1 week in culture (Figure 1(a)). They took up Di-ac-LDL (red) and were positive for FITC-UEA-1 (green) (Figure 1(b)). Immunostaining indicated cells were positive for VIII factor (Figure 1(c)), and immunophenotyping analysis revealed that expanded EPCs expressed VEGFR-2 (60.95 ± 0.88%), AC133 (16.08 ± 0.44%), CD34 (77.19 ± 3.32%), and VE-Cadherin (25.54 ± 0.73%), confirming their endothelial cell characteristics [24].

### 3.2. Ox-LDL Induces EPC Dysfunction

**Figure 2:**
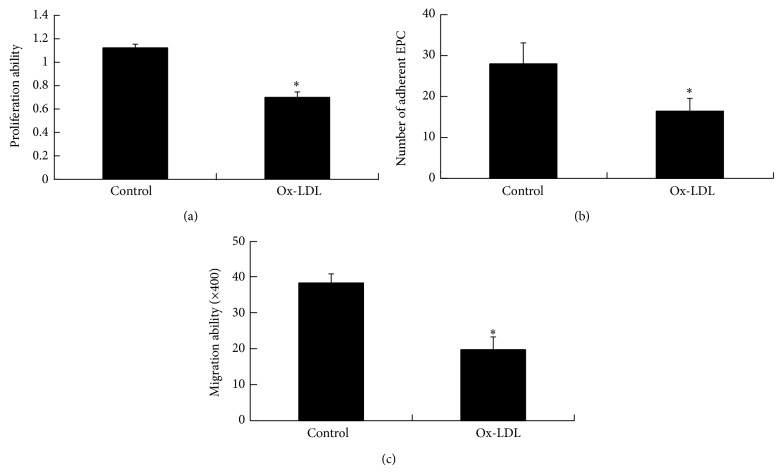
Effects of ox-LDL on EPC function. (a) Ox-LDL treatment inhibited proliferation of EPCs. EPCs were incubated in the presence or absence of ox-LDL for 24 hours as indicated. Cell proliferation was assayed by CCK8 method. (b) Ox-LDL decreased the number of adherent EPCs. EPCs were incubated in the presence or absence of ox-LDL as indicated, and adherent cells were counted. (c) Ox-LDL treatment inhibited migration of EPCs. Migration of EPCs exposed to ox-LDL was examined by transwell chemotaxis assay. Data are expressed as the means ± SEMs of triplicate experiments. ^*^ *P* < 0.01 versus control.

To evaluate the effect of ox-LDL on EPC function, we evaluated proliferation, migration, and adhesion ability of EPCs in the presence or absence of 50 *μ*g/mL ox-LDL. The results showed EPC cultures in the presence of ox-LDL contained significantly fewer EPCs after 18 hours compared with control (OD_450_: 0.700 ± 0.046 versus 1.119 ± 0.031; *P* < 0.01) (Figure 2(a)). In addition, ox-LDL significantly inhibited the adhesion activity of EPCs (Figure 2(b)). Migration of EPCs was assessed using a modified Boyden chamber in the presence or absence of ox-LDL. Again, ox-LDL significantly inhibited the migratory activity of EPCs (Figure 2(c)). Thus ox-LDL can impair function of EPCs.

### 3.3. Ox-LDL Induces ROS Accumulation of EPCs

**Figure 3:**
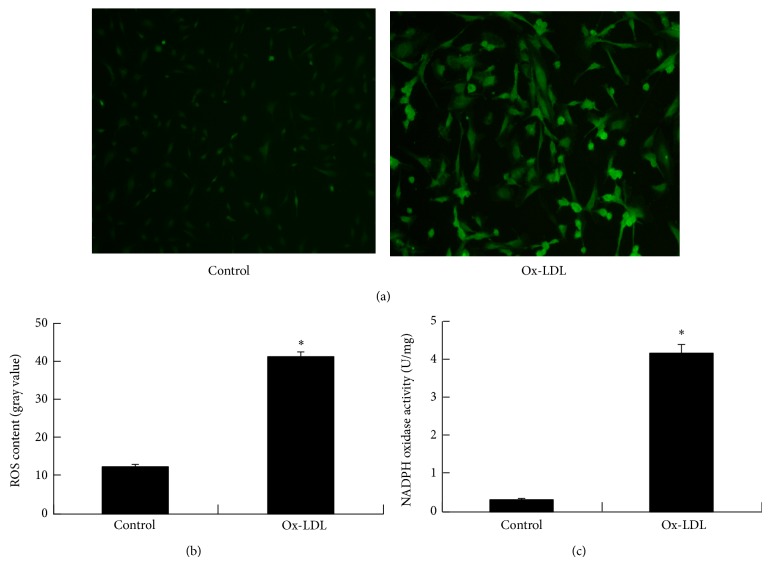
Ox-LDL induces elevation of ROS levels through increasing NADPH oxidase activity. Human EPCs isolated from peripheral blood were treated with ox-LDL. (a) Represent images of cells after culture of EPCs with ox-LDL for 1 day. Images are visualized at ×200 magnification. Oxidative stress is indicated by green fluorescent dye. ((b), (c)) Intracellular ROS generation and NADPH oxidase activity are expressed as fold over normal control. ^*^ *P* < 0.01 versus control.

ROS are well known to mediate EPC dysfunction [25]. Thus, we assessed the effects of ox-LDL on oxidative stress in EPCs. Intracellular accumulation of ROS was monitored using a fluorescent dye indicator, dichlorofluorescein diacetate. EPCs showed a significant increase of ROS after exposure to 50 *μ*g/mL of ox-LDL (12.13 ± 0.71 versus 41.22 ± 1.06, *P* < 0.01) (Figures 3(a) and 3(b)).

As NADPH oxidase is required for ROS production, we determined whether NADPH oxidase activity was regulated by ox-LDL. Assays revealed that NADPH oxidase activity was increased in response to ox-LDL treatment (4.14 ± 0.22 versus 0.32 ± 0.03, *P* < 0.01) (Figure 3(c)).

### 3.4. Ox-LDL Induces Expression and Translocation of NF-*κ*B

**Figure 4:**
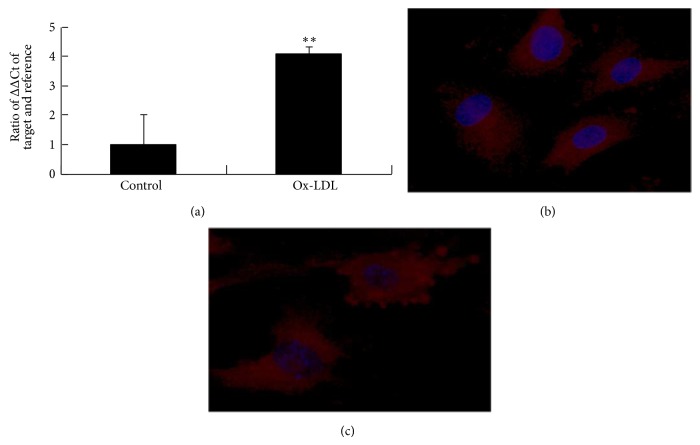
Ox-LDL affects NF-*κ*B transcription and translocation. (a) RT-PCR demonstrated that ox-LDL treatment of EPCs significantly increased the mRNA expression of P65 subunits of NF-*κ*B. ^**^ *P* < 0.05 versus control. ((b)-(c)) In untreated cells, most of the fluorescence staining for NF-*κ*B was in the cytoplasm. After cells were treated with ox-LDL, fluorescence staining for NF-*κ*B increased in the nuclei area.

Ox-LDL was then tested for its effect on NF-*κ*B transcription and translocation in EPCs. RT-PCR demonstrated that ox-LDL treatment significantly increased the mRNA levels of P65 subunits of NF-*κ*B (Figure 4(a)). NF-*κ*B translocation was then investigated by immunofluorescence staining (Figure 4(b)). In untreated cells, most of the fluorescence staining for NF-*κ*B was in the cytoplasm. After cells were treated with ox-LDL, fluorescence staining for NF-*κ*B increased in nuclei area, indicating translocation of NF-*κ*B from cytoplasm into nucleus.

## 4. Discussion

In the present study, we showed that ox-LDL induced dysfunction of EPCs in terms of proliferation, mobilization, and adhesion. In addition, ox-LDL induced expression and translocation of NF-*κ*B and oxidative stress.

Endothelial dysfunction is an initial trigger of atherosclerosis and a well-known predictor of future adverse cardiovascular events [26, 27]. Therefore, maintenance of endothelial function is important for patients with cardiovascular diseases. Recent studies have shown that the number of EPCs is a significant predictor of future cardiovascular events [28, 29]. EPCs also play a critical role in postischemic vascular repair [30, 31]. Ox-LDL is one of the most important risk factors of cardiovascular disease and has been shown to have a detrimental effect on EPC function. For example, Ma et al. found that ox-LDL decreased EPC survival and impaired its adhesive, migratory, and tube-formation capacities and further revealed that ox-LDL dose-dependently decreased Akt phosphorylation and eNOS protein expression of EPCs. Ox-LDL may cause apoptosis of EPCs via activating Bax [32] and inactivating Akt within EPCs by nitrosylation of the P85 subunit of PI3K [33]. Activation of P38 mitogen-activated protein kinase (MAPK) by ox-LDL also caused decreased survival and activity of EPCs [22]. Ox-LDL accelerates the onset of EPC senescence, which may be related to telomerase inactivation. Ox-LDL-induced EPC senescence leads to the impairment of proliferative capacity [19]. Ox-LDL level correlate with NO bioavailability in EPCs. NO can prevent EPC dysfunction in diabetic patients [34].

In the present study, we found that ox-LDL increased generation of ROS in EPCs, which is inconsistent with the previous report by Tie et al. [35]. They found that ox-LDL increased generation of H_2_O_2_ and O_2_^−^. Furthermore, we found that ox-LDL induced expression and translocation of NF-*κ*B, whether cellular oxidant stress contributes to activation of NF-*κ*B in EPCs is unclear. NF-*κ*B is an oxidant-responsive transcription factor that regulates the expression of many genes involved in inflammatory response [36]. However, the mechanism by which oxidants regulate NF-*κ*B activation has remained elusive [37]. Nevertheless, it is intriguing to hypothesize that ox-LDL causes oxidative stress and subsequent NF-*κ*B activation in EPCs.

The results of this study also demonstrated that the activation of NF-*κ*B in EPCs was associated with a functional consequence, that is, decreased proliferation, migration, and adhesion of EPCs. It is speculated that ox-LDL activates the NF-*κ*B pathway, upregulating the expression of proinflammatory cytokines involved in EPC dysfunction. Indeed, several lines of evidence suggest that extensive inflammatory stimulation may induce EPC dysfunction in humans. For example, C-reactive protein (CRP) is associated with senescence of EPCs in preeclampsia patients [38]. CRP exerts direct inhibitory effects on EPC survival [39] and angiogenic activity [40]. High levels of TNF-*α* also inhibited EPC survival [41, 42]. Finally EPCs that are mobilised in response to inflammatory stimulation may be functionally impaired [43]. Thus functional activity of EPCs is significantly impaired in the presence of inflammatory stimulation.

In conclusion, we demonstrate for the first time that ox-LDL impairs EPC function at least partly by causing oxidative stress and activating NF-*κ*B pathway. Future studies are needed to explore its underlying mechanisms. The potential benefits of antioxidants on EPC function deserve further study.
